# Blood-Letting

**Published:** 1875-10

**Authors:** M. R. May

**Affiliations:** Athens, Tennessee


### BLOOD-LETTING.

By M. R. MAY, M.D., Athens, Tenn.

*Editors Medical and Surgical Journal:*

In your last issue, under the caption of "A Lost Art," taken from the *St. Louis Record* of June, we find that the editor thanks heaven that it is a lost art.

In ancient times the sibyls and priests were placed upon the tripod to deliver oracles; but we were under the impression that an editor in his *sanctum sanctorum*, at the present time, mounts his tripod to give us facts predicated upon reason, or some basis on which conclusions might be drawn in support of his assertions. When the editor of the *St. Louis Record* congratulated himself that blood-letting was a lost art, and saw proper to hurl his philippics against the "Nestor of American surgery," because he (Dr. S. G. Gross) entertained views antipodal to his own, would it not have been in accordance with professional courtesy and legitimate discussion to have given his reasons why and wherefore he indulged in his animadversions? If blood-letting is "a lost art," the fact has not been developed to some of the most distinguished members of the medical profession, as I will endeavor to prove by citations from their works and speeches. Sir James Paget, Bart., delivered an address before the British Medical Association a short time ago, in which he advocated the use of blood-letting and gave his reasons therefor. At the same time he advised the younger members of the profession not to be deterred from its use. (*Med. Times and Gaz.*) A short time afterwards Benjamin W. Richardson, M.D., F.R.S., "remarked that the speech of Sir James Paget at Norwich on the practice of blood-letting" has created, as everything that emanates from a mind so clear, truthful and open must create, a marked effect on the fate of one of the oldest and most potent of remedies, and stated further "that in giving up blood-letting as a means of cure, it sacrifices both skill and duty to ignorant prejudice and ignoble fear, alluding to an age of placebo." (*Med. Times and Gaz.*) In a course of lectures on fever, delivered by Hudson, Physician to the Meath Hospital, page 208, he expresses himself thus: "I believe that hundreds of cases of fever have been arrested chiefly by three measures which medical fashion, rather than reason or experience, has long since consigned to comparative neglect. These are blood-letting, emetics and cold effusion.

In Chambers' lectures—Renewal of Life—page 616, he says: "It is time now to have done with the reactions for or against blood-letting, which has been going on through the period of the Christian era. The wave which has swelled backwards and forwards to a dangerous height ought to settle down in a steady stream. *We ought to know clearly why we bleed, and then we shall know when to bleed.* He goes on to give the rationale of blood-letting, and why in certain cases it meets his approbation, and when used as he recommends it, is "restorative and reconstructive," and hence not *spoliative.*

I saw in your Journal of December 1874, a communication to the *Lancet* by Dr. Rawdon McNamara on the subject of blood-letting, which goes to fortify the position of the distinguished and representative men alluded to above. Anything seen in the *Lancet* is understood by the laity as being *ex cathedra.* And even the conservative Prof. A. Flint, in his treatise on the Principles and Practice of Medicine, page 162, 2d edition, says: "*In the early period of an acute inflammation, accompanied by high fibrile movement, as indicated by a pulse accelerated and of abnormal strength, the abstraction of blood affords relief*, and may contribute to a favorable progress of the disease." In Hartshorne's Essentials, 3d edition, page 89, find the following, viz: "Now it must be admitted that blood-letting has more opponents and fewer defenders than at any previous period in medical history. Why is this? By reason of false construction and misapplication of recent science, leadership and fashion."

Now, when Prof. Gross advocated blood-letting, and it met the approbation of the distinguished members of the association as manifested by their applause, he certainly was surrounded by appreciative auditors, representative members of the profession. Suppose some one (after he had taken his seat) had whispered in his ear that he was mistaken; that blood-letting was "a lost art;" had been laid in the "catacombs of age, along with amulets, charms, and black-letter folios of the mediæval alchemists, never to be revived till "Paracelsus and Mithridates" hold high carnival, and informed the Professor that unless he retracted at once that he would be anathematized as a disciple of a character in Gil Blas—Dr. Sangrado—do you suppose it would have been to the Nestor of American surgery a subject of mortification?

We have written in no spirit of acrimoniousness. Neither because we are afflicted with *cacoethes scribendi*, neither because we write *currente calamo*, but because we considered the "art" we have so long practised had been wantonly attacked. We have not as yet been enrolled amongst the hæmapholi. And we will continue to pursue the "art" that we have found available (at least in our judgment) and encouraged by so many distinguished exemplars until influenced by statistics and an array of facts, which we have failed to see as yet, to induce a contrary course. Neither do we propose at any subsequent time, in forming our opinion, to have our judgment warped by the misconstruction of broad assertions for postulates, and strictures for facts. We will close, for fear of intruding on your time and space, both to you and the public being invaluable.

